# Delirium: A Marker of Vulnerability in Older People

**DOI:** 10.3389/fnagi.2021.626127

**Published:** 2021-04-30

**Authors:** Giuseppe Bellelli, Justin S. Brathwaite, Paolo Mazzola

**Affiliations:** ^1^School of Medicine and Surgery, University of Milano-Bicocca, Milan, Italy; ^2^Acute Geriatrics Unit, San Gerardo Hospital ASST Monza, Monza, Italy; ^3^Department of Emergency Medicine, Boston University, Boston, MA, United States

**Keywords:** delirium, elderly, frailty, Atypical symptoms, confusion

## Abstract

Delirium is an acute neuropsychiatric syndrome and one of the most common presenting symptoms of acute medical illnesses in older people. Delirium can be triggered by a single cause, but in most cases, it is multifactorial as it depends on the interaction between predisposing and precipitating factors. Delirium is highly prevalent in older patients across various settings of care and correlates with an increased risk of adverse clinical outcomes. Several pathophysiological mechanisms may contribute to its onset, including neurotransmitter imbalance, neuroinflammation, altered brain metabolism, and impaired neuronal network connectivity. Several screening and diagnostic tools for delirium exist, but they are unfortunately underutilized. Additionally, the diagnosis of delirium superimposed on dementia poses a formidable challenge – especially if dementia is severe. Non-pharmacological approaches for the prevention and multidomain interventions for the treatment of delirium are recommended, given that there is currently no robust evidence of drugs that can prevent or resolve delirium. This article aims to review the current understanding about delirium in older people. To achieve this goal, we will describe the epidemiology and outcomes of the syndrome, the pathophysiological mechanisms that are supposed to be involved, the most commonly used tools for screening and diagnosis, and prevention strategies and treatments recommended. This review is intended as a brief guide for clinicians in hospital wards to improve their knowledge and practice. At the end of the article, we propose an approach to improve the quality of care provided to older patients throughout a systematic detection of delirium.

## Introduction

Several years ago, Lipowski wrote: “Acute medical confusion as a presenting symptom holds a central position in the medicine of old age […] acute confusion is a far more common herald of the onset of physical illness in an older person, than are, for example, fever, pain or tachycardia. The elderly, especially the very old, are uniquely prone to delirium as a consequence of almost any physical illness or of intoxication with even therapeutic doses of commonly used drugs […] Failure to diagnose delirium and to identify and treat its underlying causes may have lethal consequences for the patient, since it may constitute the most prominent presenting feature of myocardial infarction, pneumonia, or some other life-threatening physical illness” (Lipowski, 1983, 1989).

Reading these sentences, the reader sees clearly how delirium has long been recognized as one of the most important components of the geriatric symptomatology, and a prognostic marker of older patients with acute illnesses. Nevertheless, in everyday practice, physicians and nurses often lack both knowledge and training about this syndrome (Bellelli et al., 2014b), referring to it with heterogeneous jargon (Teodorczuk et al., 2013). Moreover, because countless junior doctors still underestimate the impact of this condition in older people, they lack perspective about the implementation of preventative and therapeutic measures across settings of care. Finally, the lack of definite biomarkers and pharmaceutical agents specific for delirium contribute to the low impact of delirium on medical culture.

The scope of this article is to review the current understanding about delirium in older people, describing its epidemiology and outcomes, the pathophysiological mechanisms that are supposed to be involved, the most commonly used tools for screening and diagnosis, and prevention and treatments recommended. A particular focus is placed on the diagnostic challenge of delirium superimposed on dementia (DSD). We hope that this review will serve as a brief guide for clinicians working in acute hospitals to improve the quality of care provided toward their older patients.

## Definition and Causes of Delirium

The Diagnostic and Statistical Manual of Mental Disorders, Fifth Edition (DSM-5), (American Psychiatric Association, 2013) defines delirium as a neuropsychiatric syndrome that encompasses different signs and symptoms, especially disturbances in attention and awareness. This set of signs and symptoms represents the altered reaction of the CNS and its functioning when dealing with acute medical conditions, intoxication or withdrawal of medications, surgery, and electrolyte or metabolic imbalances (American Psychiatric Association, 2013). Delirium can be triggered by a single cause, but in most cases, it is multifactorial, resulting from the interaction between predisposing and precipitating factors. The higher the burden of the predisposing factors, the lower the magnitude of the precipitating factors required to cause delirium (Inouye and Charpentier, 1996; Inouye et al., 2014).

According to a landmark study by Inouye and Charpentier (1996), delirium has four main predisposing and five major precipitating factors. The former includes dementia/cognitive impairment, sensory deprivation, dehydration, and severity of an acute occurring illness, while the latter encompass poor nutritional status/malnutrition, use of physical restraints, the recent prescription of three or more new medications, urinary bladder catheterization, and other iatrogenic factors (Inouye and Charpentier, 1996). However, a number of other predisposing factors have been identified since that study. A systematic review and meta-analysis including 11 articles (total study population = 2,338 patients), identified dementia, illness severity, visual impairment, urinary catheterization, low albumin levels, and increased length of hospital stay as risk factors for delirium (Ahmed et al., 2014). Cognitive impairment and dementia were also prominent risk factors for delirium in a systematic review on patients with hip fracture and in another systematic review on patients from Intensive Care Unit (ICU) (Oh et al., 2015; Zaal et al., 2015). Recently, a study by Bowman et al. (2020) identified 55 risk factors that were predictive of delirium occurring in the community or recorded in emergency hospitalisation. These included cognitive impairment or mental illness, psychoactive drugs, frailty, infection, hyponatraemia and anticholinergic drugs (Bowman et al., 2020). Recent studies are suggesting the SARS-CoV-2 infection may be a potential cause of delirium in older people admitted to hospital wards (Zaal et al., 2015).

## Epidemiology

Delirium is common in older people across various settings of care. A recent systematic review and meta-analysis identified 33 studies that evaluated the occurrence of delirium in medical inpatients over time (Gibb et al., 2020). Only studies using internationally accepted diagnostic criteria for the diagnosis were included. Overall, delirium prevalence was 23% (95% CI 19–26%), with variations related to diagnostic criteria used (highest in DSM-IV, lowest in DSM-5), a proportion which was similar to a systematic review performed 14 years earlier (Siddiqi et al., 2006). There are no systematic reviews about its incidence in surgical patients, but studies demonstrate that delirium is a common surgical complication among older adults, with incidence of 15–25% after major elective surgery and 50% after high-risk procedures such as hip-fracture repair and cardiac surgery (Marcantonio, 2017). Delirium occurrence might be even higher in the ICU. A recent systematic review and meta-analysis including 48 studies from medical, surgical, or specialty ICUs (total number of patients = 27,342) found an overall pooled prevalence of 31% (Krewulak et al., 2018). However, in mechanically ventilated patients, delirium occurrence can range from 60 to 80% (Ely et al., 2001a).

The burden of this syndrome is also relevant in the post-acute care setting and rehabilitation facilities, where delirium prevalence is about 14–18% (Morandi et al., 2014; Bellelli et al., 2016), and in nursing homes, where delirium prevalence, according to a review, can range from 1.4 to 70% (de Lange et al., 2013). More recently, a study with 1,454 patients from 71 nursing homes, found a delirium point-prevalence of 36.8% (Morichi et al., 2018). Delirium is thought to be less frequent in the community (1–3%) (Inouye et al., 2014), but studies are limited by the methods to detect it and the selection criteria. Moreover, it should be considered that delirium onset usually leads the patient to be referred to an emergency room, a setting in which this syndrome is present in 8 to 17% of older patients, and up to 40% in nursing home residents (Inouye et al., 2014).

## Outcomes

Patients with delirium show an increased risk of developing poor clinical outcomes, including increased likelihood of nursing home placement and death (Witlox et al., 2010; Inouye et al., 2014). When experienced during a hospitalization, delirium increases 2-year mortality risk by approximately two-fold after adjusting for age, gender, chronic diseases, and dementia (Witlox et al., 2010). The negative effect of delirium on mortality has also been shown in patients with SARS-CoV-2 infection (Marengoni et al., 2020; Rebora et al., 2020). Importantly, the longer its duration, the higher the risk of death. A study performed in a group of older patients who underwent surgery after hip fracture showed that post-operative delirium incremented the hazard ratio of 6-month death by 17% per day of experiencing the syndrome, after adjusting the model for potential confounders (Bellelli et al., 2014a). Delirium may also increase the likelihood of developing cognitive impairment and/or progress to dementia. Recently, a systematic review by Goldberg et al. performed a meta-analysis of 24 studies, with 3,562 subjects experiencing delirium and 6,987 controls who did not. The authors found that delirium and the development of long-term cognitive deterioration were significantly associated (Goldberg et al., 2020). All studies demonstrated that patients who developed delirium also displayed worse cognitive performances at follow-up (Goldberg et al., 2020).

The emotional distress of patients, caregivers and healthcare workers is a further negative outcome of delirium, since it may affect not only patients, but also their family members and the members of the hospital and nursing home staff (Mc Donnell and Timmins, 2012; Morandi et al., 2015; Schmitt et al., 2019). Interestingly, a study described that these three categories (i.e., patients, family caregivers, and nurses) share three common themes of delirium-related burden, namely symptom burden, emotional, and situational burden (Schmitt et al., 2019). These findings support the theory of delirium as a shared experience, indirectly suggesting that multidisciplinary system-wide strategies may better address delirium-associated interpersonal consequences (Schmitt et al., 2019).

## Pathophysiology

The pathophysiology of delirium still remains speculative and it may represent a diverse range of pathobiology processes rather than a single entity. From an historic perspective, delirium has been viewed as a disorder of several neurotransmitters, including acetylcholine, melatonin, dopamine, norepinephrine, glutamate, 5-hydroxytryptamine or serotonin, histamine, and/or gamma-aminobutyric acid (Maldonado, 2018). However, the pathophysiology of delirium is more complex than that. Here, we present only some of the most promising current theories on this topic. One theory proposes that delirium is the result of the combination of disorders in the neurotransmission, a failure in integrating and processing sensory signals and motor effectors, and a breakdown in cerebral network connectivity (Maldonado, 2018). It postulates that several patient-specific factors interact to develop delirium, including neuroinflammation, excess of “oxidative stress,” “neuroendocrine dysfunction” and “circadian rhythm or melatonin dysregulation” (Maldonado, 2018). For example, a peripheral infection or surgery may activate inflammatory cytokines and other mediators in the blood that can cross the blood brain barrier or reach the brain parenchyma through other routes (e.g., the vague nerve), and here activate the microglia cells and astrocytes (Wilson et al., 2020). The alterations of the body composition (i.e., a reduction of the lean mass and an increase of the fat mass) that commonly occur in older individuals during their life, might also play a role as additional source of inflammatory stimulus (through endocrine secretion of pro-inflammatory adipokines), amplifying the magnitude of the response (Bellelli et al., 2017).

This sequence of events leads to a misalignment in neuronal function, synaptic impairment, and the subsequent onset of the multi-faceted symptomatology of delirium, which involves neurological deficits, and alterations in the behavior and cognition. Importantly, neuroendocrine dysfunctions, excess of oxidative stressors and defect of melatonin can also contribute to neuroinflammation of the brain (Cunningham et al., 2009; Cerejeira et al., 2012), thus indirectly maintaining the synaptic dysfunction.

More recently, other mechanisms underpinning delirium onset have been proposed. The brain bioenergetics insufficiency is one of them. Neurons and astrocytes both require massive amount of glucose supplied by the microvasculature to generate adenosine triphosphate (ATP) by glycolysis (Wilson et al., 2020). However, several acute illnesses may impair the supply of glucose to the brain. For instance, lung infections can cause hypoxemia, reducing neuronal energy metabolism through an impaired mitochondrial production of ATP (Wilson et al., 2020), and hemodynamic shock can impair blood flow to the brain and thus impair glucose supply. Moreover, even microcapillary and neurovascular dysfunctions, as well as systemic hypoglycemia can all provoke insufficient glucose supply and, thus, delirium and coma (Wilson et al., 2020). Evidence to support this hypothesis comes from studies on hip fracture and medical patients with delirium (Caplan et al., 2010; Kealy et al., 2020). However, studies with Fluorodeoxyglucose – Positron Emission Tomography (FDG-PET) imaging in mice and humans have shown that glucose uptake is substantially reduced with sepsis and overall glucose metabolism is impaired during delirium (Semmler et al., 2008; Hölscher, 2019).

According to the first theory, delirium is the final product of a breakdown in the efficiency of brain network that cannot compensate for injury and diseases. For instance, in older individuals with high-grade neurodegeneration, developing insults and stressors may lead to a derangement of function between brain areas and thus to delirium (Shafi et al., 2017). This mechanism is supported by studies showing that delirious patients display atrophy in the amygdala and decreased gray matter volumes in some brain areas (Rolandi et al., 2018), that delirium duration is increased in individuals with loss of integrity in the inter-hemispheric corpus callosum (Morandi et al., 2012b), and that abnormalities in the hippocampus, thalamus, basal forebrain and cerebellum are associated with incidence and severity of delirium (Cavallari et al., 2016).

**FIGURE 1:**
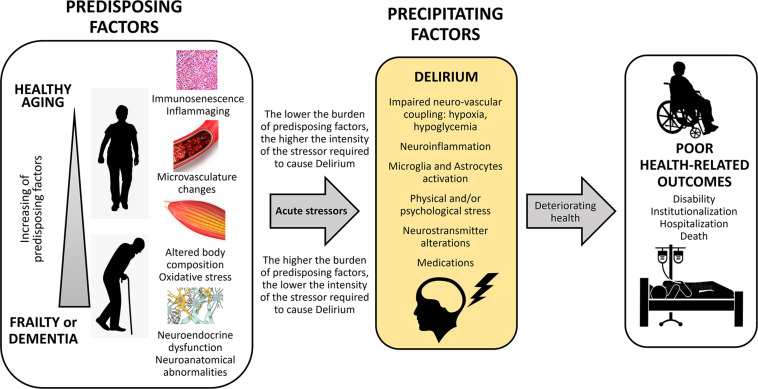
Conceptual framework of predisposing and precipitating factors leading to delirium after an acute stressor, and hesitating in poor health-related outcomes.

Figure 1 summarizes the interaction between predisposing and precipitating factors leading to delirium and to some adverse clinical outcomes.

## Diagnosis of Delirium

### Clinical Features of Delirium

Delirium is recognized by a constellation of symptoms. Inattention, disorganized thought, altered consciousness and other multiple cognitive domains represent the key features (American Psychiatric Association, 2013). However, hallucinations, delusions, incoherent speech, inappropriate behavior, emotional lability, and alterations of the sleep–wake cycle can also be present (Inouye et al., 2014).

There are at least three psychomotor subtypes of delirium, i.e., hypoactive, hyperactive, and mixed. The former is characterized by a withdrawal from interaction with the outside world, sluggishness (or lethargy) and reduced psychomotor activity; the hyperactive subtype presents restlessness, agitated behavior and/or aggressiveness; and the mixed subtype is characterized by the transition from hyperactivity to hypoactivity or vice versa. Meagher et al. (2014) described a non-hyperactive-non-hypoactive subtype of delirium, which is characterized by normal level of psychomotor activity and no fluctuations between hyperactive and hypoactive subtypes. It is still unclear which of these motor subtypes may underlie different causes of delirium. However, various studies suggest that the hypoactive and mixed subtypes are more likely to develop in frail older individuals, thus correlating with a more severe prognosis (Bellelli et al., 2007, 2018). More importantly, different psychomotor subtypes may have distinct risk factors, indirectly suggesting that they should be different clinical phenomena (Morandi et al., 2017). A potential role for altered noradrenergic activity in influencing the arousal level and therefore the delirium psychomotor subtype (Hahn et al., 1995; Matthews et al., 2002) is a longstanding object of speculation. However, further studies are needed to clarify this issue.

### Diagnostic Criteria and Screening Tools

Delirium is essentially a clinical diagnosis. Currently, the DSM-5th edition and the International Statistical Classification of Diseases and Related Health Problems, 10th revision criteria represent the gold standard for the diagnosis (Table 1). However, a number of screening tools for delirium have been developed in recent years to help physicians in its detection. Here, we will report two of the most commonly used.

**TABLE 1:** The criteria used to diagnose delirium: the Diagnostic and Statistical Manual of Mental Disorders (DSM) -5th edition and the International Statistical Classification of Diseases and Related Health Problems (ICD), 10th revision criteria.

**DSM 5th**		**ICD 10th**	
A	A disturbance in attention (i.e., reduced ability to direct, focus, sustain, and shift attention) and awareness (reduced orientation to the environment).	A	Clouding of consciousness, i.e., reduced clarity of awareness of the environment, with reduced ability to focus, sustain, or shift attention.
B	The disturbance develops over a short period of time (usually hours to a few days), represents a change from baseline attention and awareness, and tends to fluctuate in severity during the course of a day.	B	Disturbance of cognition, manifest by both:
			impairment of immediate recall and recent memory, with relatively intact remote memory;
			disorientation in time, place or person.
C	An additional disturbance in cognition (e.g., memory deficit, disorientation, language, visuospatial ability, or perception).	C	At least one of the following psychomotor disturbances:
			rapid, unpredictable shifts from hypoactivity to hyperactivity;
			increased reaction time;
			increased or decreased flow of speech;
			enhanced startle reaction.
D	The disturbances in Criteria A and C are not better explained by another pre-existing, established, or evolving neurocognitive disorder and do not occur in the context of a severely reduced level of arousal, such as coma.	D	Disturbance of sleep or the sleep-wake cycle, manifest by at least one of the following:
			insomnia, which in severe cases may involve total sleep loss, with or without daytime drowsiness, or reversal of the sleep-wake cycle; nocturnal worsening of symptoms;
			disturbing dreams and nightmares which may continue as hallucinations or
			illusions after awakening.
E	There is evidence from the history, physical examination, or laboratory findings that the disturbance is a direct physiological consequence of another medical condition, substance intoxication or withdrawal (i.e., due to a drug of abuse or to a medication), or exposure to a toxin, or is due to multiple etiologies.	E	Rapid onset and fluctuations of the symptoms over the course of the day.
	Specify whether Substance intoxication delirium: This diagnosis should be made instead of substance intoxication when the symptoms in Criteria A and C predominate in the clinical picture and when they are sufficiently severe to warrant clinical attention.	F	Objective evidence from history, physical and neurological examination or laboratory tests of an underlying cerebral or systemic disease (other than psychoactive substance-related) that can be presumed to be responsible for the clinical manifestations in A-D.
			Comments
			Emotional disturbances such as depression, anxiety or fear, irritability, euphoria, apathy or wondering perplexity, disturbances of perception (illusions or hallucinations, often visual) and transient delusions are typical but are not specific indications for the diagnosis.

The Confusion Assessment Methods (CAM) has been developed by Inouye et al. (1990). It includes an algorithm based on 4 core features of delirium: (1) acute change or fluctuating course, (2) inattention, and either or both (3) disorganized thinking, and (4) alteration of consciousness. The CAM has been validated in a number of high-quality studies showing high sensitivity (94–100%), and specificity (89–95%). There is also a CAM-ICU version for use with non-verbal mechanically ventilated patients (Ely et al., 2001b). However, preliminary training is critical for its use. In fact, one study has shown that without preliminary training of the examiners, the rate of underdiagnoses was unacceptably high (Inouye et al., 2001). Sensory deficits, dementia, the hypoactive psychomotor subtype of delirium and old age were recognized as risk factors for the under-recognition (Inouye et al., 2001).

The 4AT test is a relatively new tool proposed by Maclullich and colleagues (available at^1^). Importantly, the test does not require special training and generally takes less than 2 min to be completed; furthermore, it can be performed also in subjects with visual or hearing impairment and in “untestable” individuals with severe agitation or drowsiness (Bellelli et al., 2015a). The 4AT encompasses four items: alertness (item 1), the Abbreviated Mental Test – 4 (AMT4, item 2), attention (tested with months of the year backwards, MOTYB, item 3), acute change in mental status or its fluctuation (item 4). Recently, a prospective randomized multicenter study demonstrated that the 4AT displays a higher sensitivity compared to the CAM (76% [95% CI 61–87%] vs. 40% [95% CI 26–57%], respectively) (Shenkin et al., 2019). Conversely, the CAM had a higher specificity (100% [95% CI 98–100%] for CAM vs. 94% [95% CI 92–97%] for 4AT, respectively) (Shenkin et al., 2019). The results of this study suggest that 4AT may be proposed as a tool to improve detection of delirium as well as CAM, or that either tool may have a role in screening depending on the setting and purpose.

### Barriers to Delirium Recognition

As already mentioned, delirium has been recognized for at least two millennia as a dangerous condition, but still remains underdiagnosed. There are many possible explanations for this. One is that medical culture does not regard delirium as an important topic. For instance, many Academic courses for undergraduates, medical doctors, and nurses do not include delirium in their programs. Additionally, the medical textbooks are usually arranged by disorders while disregard syndromes like delirium. Another reason is that delirium has received various clinical labels such as “acute confusion,” “acute organic brain syndrome,” “brain failure,” “intensive care psychosis,” “toxic encephalopathy.” The use of these terms actually impede the communication among clinicians and healthcare professionals (Hall et al., 2012) and do not help promoting the knowledge of delirium. A recent statement of ten scientific societies and a review article proposed to update the nomenclature of delirium and acute encephalopathy, two commonly used terms to label the same phenomenon, to integrate them within a single framework, harmonize research efforts and advance clinical practice. According to this nomenclature, delirium should be considered a clinical syndrome while acute encephalopathy can be defined as a rapidly developing (usually within hours to a few days), diffuse pathobiological process that might manifest as delirium or, in cases of severely decreased levels of consciousness, as coma (Oldham and Holloway, 2020; Slooter and Stevens, 2020).

Another reason why delirium is undervalued is that, for many healthcare professionals, patients experiencing delirium may be only “agitated.” It is possible that the concept of delirium somewhat recalls the condition known as “delirium tremens,” which relates to alcohol abuse. But it is even more likely that the behaviors often associated with delirium, particularly agitation and aggression, causes distress and anxiety for nurses and families who then bring it to the attention of healthcare professionals. In a prospective Italian multicenter study where physicians diagnosed delirium according to their personal experience, delirium was coded in 2.9% of 2,521 older hospitalized patients, while combined deficits in attention, orientation, and memory (potentially suggestive of delirium) were found in 19.8% of patients (Bellelli et al., 2015b). Interestingly, the proportion of patients coded with delirium was similar to the proportion of patients who actually had hyperactive delirium in another Italian multicenter study, conducted on similar patients (Morandi et al., 2017). These findings suggest that, at least in Italy, physicians tend to diagnose delirium mainly when it is hyperactive. Thus, appropriate education and increasing broaden recognition of delirium syndrome is warranted.

### The Challenging Diagnosis of Delirium Superimposed on Dementia

Delirium superimposed on dementia is a common condition in older people (Fick et al., 2002, 2013) but a real diagnostic challenge for clinicians, given the relative lack of tools specifically designed for its recognition. Indeed, the DSM-5 does not provide specific indications on how to diagnose delirium when overlapping on pre-existing dementia (American Psychiatric Association, 2013) and the CAM has shown only moderate sensitivity (77%) in detecting DSD (Morandi et al., 2012a). Other tools, such as the 4AT, have never been specifically tested in DSD patients (Jeong et al., 2020). The suboptimal performance of CAM and other existing tools to detect DSD mainly lies on the fact that people with dementia may already have impairments in cognitive functions, and especially in attention, which is a key feature of delirium (Marra et al., 2018). However, attention may be impaired in patients with severe dementia, limiting the ability of the attentional tests to discriminate between delirium and dementia (O’Halloran et al., 2014; Robertson et al., 2014; Bellelli et al., 2019). Thus, selecting the right testing is crucial. In 234 older patients admitted to an Emergency Department, Marra et al. found that MOTYB had very good sensitivities but had modest specificities for delirium, limiting their use as a standalone assessment, while reciting the days of the week backwards (DOWB) had the best combination of sensitivity and specificity (Marra et al., 2018). Nearly one third of their delirious patients had also dementia (Marra et al., 2018). In a prospective cohort study of patients admitted to an acute geriatric ward, Bellelli et al. (2019) assessed 89 patients, of whom 42 were frail and 29 had dementia. Patients were all assessed with three tests (i.e., the MOTYB, the DOWB and counting backwards from 20 to 1), which showed a similar predictive capacity to detect delirium in patients with frailty and dementia (Bellelli et al., 2019); further studies confirmed the association between frailty and attentional performances (O’Halloran et al., 2014; Robertson et al., 2014).

Recently, Steensma et al. (2019) proposed a brief screening test that included 3 items: (a) listing the days of the week, from Sunday to Monday, backwards; (b) asking the patient if he/she recognizes the type of place where he/she is currently located; and (c) report whether the patient appears sleepy. Among 391 older adults with dementia, they showed that the test had excellent sensitivity (94%) but limited specificity (42%) in recognizing DSD (Steensma et al., 2019).

Another key element to detect DSD is the patient’s arousal, which is not usually impaired in dementia, even in the advanced stages. Therefore, in patients with impaired arousal (above the level of coma), the inability to engage in cognitive testing is considered severe inattention and thus a proxy of delirium (European Delirium Association and American Delirium Society, 2014). To evaluate the suitability of the tests assessing arousal for diagnosing DSD, a multicenter study recruited 645 patients with pre-existing dementia and measured their arousal’s levels with the Richmond Agitation Screening Scale (RASS) and the modified RASS (m-RASS) (Ely et al., 2003; Chester et al., 2012). Overall, a score other than 0 at the two scales (i.e., the RASS and the m-RASS) was 70.5% sensitive and 84.8% specific for a diagnosis of DSD. Using a RASS/m-RASS value greater than + 1 or less than −1 as a cut-off, the sensitivity was 30.6% (CI 25.9–35.2%) and the specificity was 95.5% (CI 93.1−98.0%) (Morandi et al., 2016). The Observational Scale of Level of Alertness (OSLA) is another tool to evaluate the patient’s arousal, and assesses patient’s eye opening, eye contact, posture, movement, and communication showing to identify delirium specifically (Tieges et al., 2013). Recently, a European multicenter study recruited 114 patients with dementia alone, delirium alone, DSD or none, hypothesizing that a combined arousal and attention testing procedure would be accurate to detect DSD (Richardson et al., 2017). Using OSLA, 83% participants were correctly classified as having delirium while the attention test 76% of participants. However, combining scores correctly classified 91% of participants with delirium (sensitivity 84%, specificity 92%) and diagnostic accuracy remained high in the patients with dementia (sensitivity 94%, specificity 92%) (Richardson et al., 2017).

An additional observation is that delirium originating in a context of pre-existing cognitive impairment does not alter only the cognitive status but can affect the motor functions as well (Bellelli et al., 2011; Gual et al., 2019). Indeed, one case-controlled study with prospective evaluations of four groups of patients with delirium alone, dementia alone, DSD or none, demonstrated that patients with delirium had fluctuations of motor performance (as assessed with the Trunk Control test) (Franchignoni et al., 1997) that were chronologically related with the onset of delirium, a pattern that was especially apparent in patients with DSD (Bellelli et al., 2011). Importantly, patients with DSD had severe pre-existing dementia (Bellelli et al., 2011). A more recent cross-sectional multicenter study recruiting 114 consecutive patients and measuring function with the Hierarchical Assessment of Balance and Mobility (HABAM) score (MacKnight and Rockwood, 1995), confirmed that individuals with delirium have worse motor function than those without delirium, especially if they had comorbid dementia (Gual et al., 2019). Pathophysiological explanations of these findings may include the acute imbalance of the cerebral brain networks connectivity that may occur during delirium (Maldonado, 2018).

**FIGURE 2:**
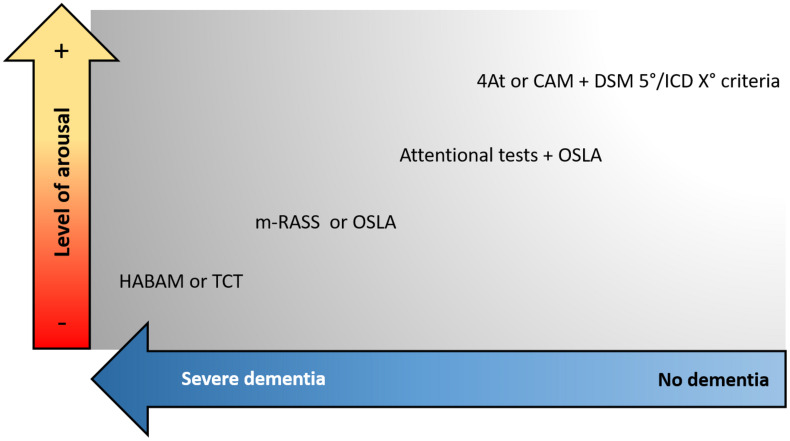
Proposed approach to select the screening tools for delirium according to the presence of dementia and the patient’s level of arousal. CAM, Confusion Assessment Method; DSM, Diagnostic and Statistical Manual of Mental Disorders; ICD, International Classification of Diseases; OSLA, Observational Scale of Level of Arousal; m-RASS, modified Richmond Agitation and Sedation Scale; HABAM, hierarchical assessment of balance and mobility; TCT, Trunk Control Test.

We suggest that the assessment of motor functions can be helpful to detect DSD, especially in those patients with severe pre-existing dementia that are difficult to approach with verbal communication. A scheme for selecting the tools to assess delirium according to the severity of dementia and level of arousal is proposed in Figure 2.

## Prevention

### Non-pharmacological Approaches

There is robust evidence that non-pharmacological approaches are the best way to prevent delirium in hospitalized patients. In 1999, Inouye and colleagues described the hospital Elder Life Program (HELP), a model of care tailored for older patients and specifically oriented to prevent delirium during the hospital stay (Inouye et al., 1999). Eight hundred and fifty-two inpatients aged (≥70 years were enrolled, and the investigators carefully administered and tracked standardized interventions to manage six risk factors, assessed on admission. Those included vision impairment, hearing impairment, sleep deprivation, cognition, dehydration, and immobility. The intervention strategies included therapeutic activities, limited use of psychoactive drugs, reorientation, promotion of sleep, maintenance of adequate hydration and nutrition, early mobilization, and provision of visual and hearing adaptations. An experienced interdisciplinary team provides the HELP, with the assistance of trained nurses or volunteers. Overall, the authors found significant decrease in the incidence (number of episodes) and duration of delirium after applying this multicomponent program, suggesting that primary prevention may represent the most effective approach for treating this syndrome (Inouye et al., 1999). After this seminal work, the program has been replicated in other sites after being adapted (Inouye et al., 2000), and several systematic reviews and meta-analyses have assessed the efficacy of multicomponent non-pharmacological approach in preventing delirium. One systematic review and meta-analysis identified 14 high-quality trials for a total number of 4,267 patients, finding that a bundle of non-pharmacological and multicomponent interventions decreased the incidence of delirium by 44% (OR, 0.56; 95%CI, 0.42–0.76) (Hshieh et al., 2015). Importantly, this approach leads to a reduction of the rate of falls (Hshieh et al., 2015). Recent guidelines from Scientific Associations and Cochrane reviews incorporated these recommendations (Siddiqi et al., 2016; SIGN, 2019). The presence and involvement of family members at the patient’s bedside has also shown potential efficacy in reducing the incidence of post-operative delirium and the rate of cognitive and physical deterioration at discharge (Wang et al., 2019). More recently, a systematic review integrated the evidence of the multicomponent interventions in ICU and non-ICU settings, confirming the current guidelines that non-pharmacological interventions are effective in preventing delirium, with a global risk ratio = 0.53 (95% CI = 0.41–0.69) (Ludolph et al., 2020).

Non-pharmacological approaches recommended by reviews and guidelines include the use of reorientation strategies (e.g., orientation boards, calendars, clocks), the promotion of patient’s hydration, sleep, mobilization, and the use of assistive devices such as eyeglasses and hearing aids, if needed. Physical restraints should be avoided due to their role in worsening agitation and increasing risk of strangulation (Inouye et al., 2007). Another important element of non-pharmacological prevention includes the optimization of pain control with non-opioids and avoiding high-risk medications, such as those with anticholinergic effect (National Clinical Guideline Centre, 2010; SIGN, 2019).

Overall, despite strong evidence supporting their value, the implementation of delirium preventive measures is far from being the rule in most hospitals. Main barriers to implementation include the time constraints of the staff and cultural gaps that are widely diffused among physicians and nurses (Greysen, 2015). Furthermore, modifying the everyday practice in the acute hospital setting is difficult and is often perceived as risky and fraught with uncertainty, especially regarding the true benefits of the changing process for both patients and the healthcare system (Greysen, 2015). Future efforts are thus required to increase the rate of implementation of multicomponent non-pharmacological preventive measures of delirium, rather than pharmacological ones.

### Pharmacological Approaches

The idea to prevent delirium using pharmacological interventions is fascinating but, at the present, poorly supported by the literature. Use of antipsychotics has been investigated, given their efficacy in psychiatric diseases. However, this approach is ineffective if not potentially harmful. Two recent systematic reviews evaluating the effect of first- and second-generation antipsychotics over placebo found no difference in delirium incidence, its duration, in the length of hospital stay, and in mortality among groups (Neufeld et al., 2016; Oh et al., 2019). Furthermore, some trials showed that the use of antipsychotics is associated with a higher occurrence of potentially detrimental cardiac effects (Oh et al., 2019). Benzodiazepines are similarly ineffective and may cause harm due to sedation, and should therefore be avoided, except in alcohol or benzodiazepine withdrawal-related delirium (SIGN, 2019).

There may be some emerging promising evidence for melatonin and its endogenous hormone. A recent network meta-analysis of six randomized controlled trials demonstrated significant preventive effects with melatonin (both at 5 and 0.5 mg/day) and ramelteon (8 mg/day) against placebo groups (Yang et al., 2020). Furthermore, in a recent multicentre randomized placebo-controlled trial, Hatta et al. showed that suvorexant (used in the treatment of insomnia), every night for 3 days in 72 hospitalized older patients, significantly reduced the occurrence of delirium compared to the administration of placebo (Hatta et al., 2017). A meta-analysis that included seven studies conducted in patients undergoing this treatment compared with controls (402 treatment patients and 487 controls) showed that delirium incidence could be markedly reduced (Odds Ratio, 0.30; *P* < 0.001) and time to delirium onset was significantly lengthened in the treatment groups compared to controls (Xu et al., 2020). Further larger studies are required.

## Treatment

### Non-pharmacological Approaches

There are several guidelines from scientific and academic societies that offer guidance and practical recommendations in the management of delirium in older patients (National Clinical Guideline Centre, 2010; SIGN, 2019).

Once a patient is recognized with delirium, it is first important to identify acute and life-threatening causal factors, including hypotension, low tissue oxygenation, drug overdose or withdrawal, and hypoglycemia. Once life-threatening conditions have been corrected, the second step is the identification of the underlying causes of delirium. To help this task, some mnemonics have been proposed (Marcantonio, 2017). They may be particularly useful given that delirium has commonly a multiple etiology and the identification of only one cause may be insufficient, leading to inappropriate management of the patient (Ferrara et al., 2019). The third step is the adoption of non-pharmacological treatments. Unnecessary tubes, catheters and physical restraints should be avoided; conversely, approaching patients at the bedside with recognizable faces and adopting tools to facilitate orientation (e.g., calendar, clock) are useful to improve restlessness and mild-to-moderate agitation. It is also recommended to promote sleep hygiene and optimize nutritional and fluid intake (SIGN, 2019). Another potential target of treatment is the environment. Unfamiliar environment, excessive noise and ward moves may indeed be precipitating factors of delirium (Van Rompaey et al., 2012). It is also important to actively involve families (Rosa et al., 2017) and multidisciplinary professionals (such as occupational therapists) in care delivery, to improve mobility and participation (Pozzi et al., 2017).

### Pharmacological Approaches

Delirium presenting with severe agitation (or combativeness) requires immediate non-verbal and verbal de-escalation techniques. If this approach fails, if agitation is severe and stressful for the patient and/or can endanger the provision of life-sustaining therapies, a pharmacological approach should be considered (National Clinical Guideline Centre, 2010). However, pharmacological agents result in sedation and may perpetuate delirium. Other steps must also be considered.

First, it is crucial to keep in mind that any changes in medications, including over-the-counter and herbal medications, or changes in dosage of medication or abrupt withdrawal of medication could result in delirium. Benzodiazepines, opiates, tricyclic antidepressants, anticholinergic medications, antihistamines and tramadol should be avoided or reduced if possible (SIGN, 2019). Constipation and urinary retention are common causes of severe agitation in people with moderate to severe dementia, and therefore, a systematic exclusion of their presence is required. If pain is likely, e.g., due to trauma or painful procedures (e.g., surgery), or causes of delirium are not identified in a patient with moderate to severe dementia, analgesics should be initiated (Sampson et al., 2020). In fact, characteristic fluctuations in attention and awareness may affect the capability of self-reporting pain, making its recognition, assessment and treatment more difficult (Sampson et al., 2020). If analgesic treatment is required, non-opioid agents may be preferable, while the opioid with the lowest odds of causing delirium is oxycodone (SIGN, 2019).

The choice of antipsychotic medications in hyperactive delirium is object of debate, and recent systematic reviews have been published (Nikooie et al., 2019; Rivière et al., 2019). Rivière et al. investigated the efficacy and tolerability of atypical antipsychotics, finding some evidence that quetiapine and olanzapine are suitable alternatives to haloperidol (Rivière et al., 2019). However, the authors found high heterogeneity among studies that prevented them to perform a meta-analysis. Nikooie et al. extracted data from 16 randomized controlled trials and 10 observational studies of antipsychotic vs. another antipsychotic or antipsychotic vs. placebo, without finding differences and concluding that the routine administration of haloperidol or second-generation atypical antipsychotics is not supported by the current evidence (Nikooie et al., 2019). Again, the authors found wide heterogeneity in the dosage of antipsychotics, outcomes and measurement tools among studies (Nikooie et al., 2019).

A different approach is required if delirium is due to alcohol withdrawal, if there is a contraindication to antipsychotics (e.g., in neuroleptic malignant syndrome or Parkinson’s disease), if the patient has dementia with Lewy bodies or for acute seizure management. In these cases, benzodiazepines should be considered (National Clinical Guideline Centre, 2010). Another case in which benzodiazepines may be used is the hyperactive delirium in patients with advanced cancer. One study performed among patients affected by advanced cancer focusing on the end-of-life period (last weeks or days of life), showed a reduction in agitated behavior by adding a single 3 mg dose of lorazepam intravenously to haloperidol, when compared with placebo (Hui et al., 2017). However, this treatment option should be considered for refractory symptoms only (after antipsychotics have failed), given that findings come from a small study, using a single dose of lorazepam.

**FIGURE 3:**
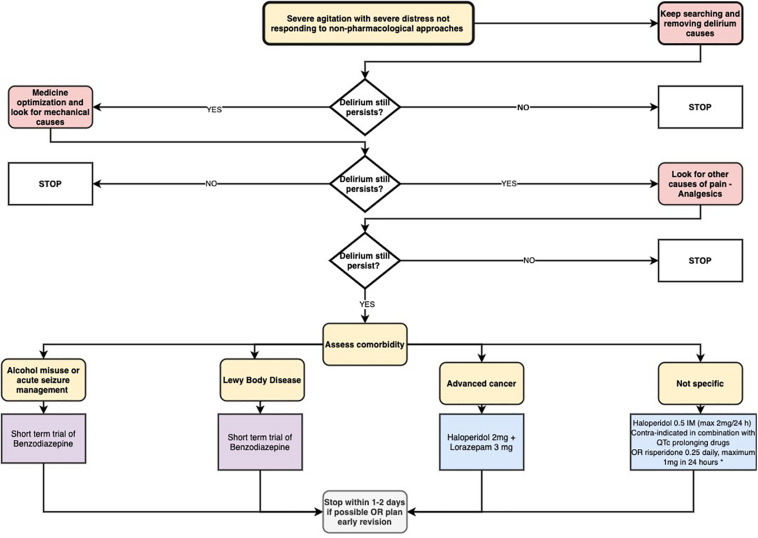
Proposed pharmacological approach for the agitated delirium (Delirium: diagnosis, prevention, and management. NICE clinical guideline n. 103, 2010). * The single study supporting this indication was restricted to patients with advanced cancer in end-of-life care. Please note that this treatment option should be considered for refractory symptoms only (after antipsychotics have failed).

Figure 3 summarizes the proposed pharmacological approach for the agitated delirium.

## Future Perspectives and Summary

As of now, the medical community still does not recognize delirium as it should, and this remains an international problem of immense gravity (Han et al., 2009; Erden Aki et al., 2014; Lange et al., 2019), which ultimately translates to improperly trained healthcare staff. Physicians may not actively search for the presence of delirium in their usual practice because they consider this task as superfluous and time expensive. On the contrary, we propose the systematic assessment of delirium as an opportunity for the clinicians to improve the clinical diagnostic process and thus the quality of care provided to their older patients. Here are reported the steps of this approach:

1. Because delirium is a frequent atypical presentation of diseases in the geriatric population, physicians should systematically screen it in their patients.
2. Given that delirium and its duration are associated with negative outcomes, delirium detection (and thus treatment) may be regarded as a clinical priority.
3. Once delirium has been detected, active searching for its underlying causes should be undertaken.
4. The tracking for the presence of delirium should be undertaken at least daily in acute hospital wards.
5. If, after treatment, delirium resolves, this may be an indirect sign that patient is clinically improving. Prolonged/persistent delirium may also be present despite the extensive diagnostic approach and therapeutic measures, indicating higher vulnerability or even a pathway to short-term or long-term cognitive impairment.

In other words, we propose to look at delirium as a marker of clinical instability and as a “*litmus test*” of the effectiveness of the care provided.

To summarize, delirium is a common and dangerous condition for older adults. Significant steps forward have been made regarding the understanding of pathophysiology and diagnosis, but still remain unresolved many aspects. Particularly challenging is the recognition and diagnosis of delirium superimposed on dementia. Prevention with multicomponent non-pharmacological approaches as well as non-pharmacological treatments are particularly important. Currently there are no drugs recommended for both prevention and treatment. Finally, the systematic detection of delirium is proposed as an opportunity for the clinicians to improve their clinical diagnostic process and thus the quality of care provided to their older patients.

## Author Contributions

GB, JB, and PM contributed to the conception and design of this work and drafted the manuscript. JB revised the manuscript critically for language and important intellectual content. All authors approved the final version of this work, and agreed to be accountable for all aspects of the work in ensuring that questions related to its accuracy or integrity of any part of the work are appropriately investigated or resolved.

## Conflict of Interest

The authors declare that the research was conducted in the absence of any commercial or financial relationships that could be construed as a potential conflict of interest.

## Footnotes

1 www.the4at.com
